# Late Onset of Antiretroviral Therapy in Adults Living with HIV in an Urban Area in Brazil: Prevalence and Risk Factors

**DOI:** 10.1155/2019/5165313

**Published:** 2019-04-07

**Authors:** Priscila Ribeiro Guimarães Pacheco, Ana Laura Sene Amâncio Zara, Luiz Carlos Silva e Souza, Marília Dalva Turchi

**Affiliations:** ^1^Federal University of Goias, Institute of Tropical Pathology and Public Health, Brazil; ^2^Hospital of Tropical Diseases Dr. Anuar Auad, Health Secretary of Goias State, Brazil

## Abstract

**Introduction:**

Highly active antiretroviral therapy has been available since 1996. Early initiation of antiretroviral therapy (ART) leads to improved therapeutic response and reduced HIV transmission. However, a significant number of people living with HIV (PLHIV) still start treatment late.

**Objective:**

This study aimed to analyze characteristics and factors associated with late initiation of ART among HIV-infected treatment-naïve patients.

**Methods:**

This cross-sectional study included PLHIV older than 17 years who initiated ART at two public health facilities from 2009 to 2012, in a city located in Midwestern Brazil. Pregnant women were excluded. Data were collected from medical records, antiviral dispensing forms, and the Logistics Control of Medications System (SICLOM) of the Brazilian Ministry of Health. Late initiation of ART was defined as CD4+ cell count < 200 cells/mm^3^ or presence of AIDS-defining illness. Uni- and multivariate analysis were performed to evaluate associated factors for late ARV using SPSS®, version 21. The significance level was set at p<0.05.

**Results:**

1,141 individuals were included, with a median age of 41 years, and 69.1% were male. The prevalence of late initiation of ART was 55.8% (95%CI: 52.9-58.7). The more common opportunistic infections at ART initiation were pneumocystosis, cerebral toxoplasmosis, tuberculosis, and histoplasmosis. Overall, 38.8% of patients had HIV viral load equal to or greater than 100,000 copies/mL. Late onset of ART was associated with higher mortality. After logistic regression, factors shown to be associated with late initiation of ARV were low education level, sexual orientation, high baseline viral load, place of residence outside metropolitan area, and concomitant infection with hepatitis B virus.

**Conclusion:**

These results revealed the need to increase early treatment of HIV infection, focusing especially on groups of people who are more socially vulnerable or have lower self-perceived risk.

## 1. Introduction

The benefits of highly active antiretroviral therapy (ART) have been widely demonstrated, especially when it comes to reducing morbidity and mortality, improving the quality of life of people living with HIV (PLHIV), and reducing HIV transmission [1, 2]. Prevention of mother-to-child transmission of HIV has been very successful around the world and some countries have already eliminated HIV vertical transmission [3]. Despite these major achievements, progress in reducing new sexually or intravenously transmitted HIV infections has been slow in many regions [1].

The goal of ending the HIV epidemic by 2030, regarded as a public health issue, relies on several aspects, including timely diagnosis, early initiation of ART, high adherence to treatment, and sustained undetectable viral load for the majority of HIV-infected individuals. The Joint United Nations Program on HIV/AIDS set the 90-90-90 targets to be achieved by 2020, in order to end the HIV epidemic worldwide. These targets established that 90% of all people living with HIV should know their diagnosis; 90% of all people with diagnosed HIV infection should be on ART; and 90% of all people receiving ART should achieve viral suppression [4].

The distinction between early and late treatment of HIV infection is based on CD4+ cell count level and presence of symptoms related to HIV-induced immunosuppression at treatment initiation. Late presentation of PLHIV to health care facilities and consequent late initiation of ART are associated with major medical and societal consequences [5]. Delayed therapy is associated with worse immune reconstitution [6, 7], higher frequency of opportunistic infections [8], increased morbidity [9], increased mortality [10], higher risk of cardiovascular and metabolic diseases [11], and higher risk and costs of noninfectious comorbidities [12]. From a societal perspective, late initiation of ART leads to more complex therapeutic regimens and increased costs [13, 14]. Moreover, it contributes to HIV transmission since late initiators of ART have high circulating viral load for longer periods of time [15].

Despite global efforts to control the HIV epidemic, delayed treatment of HIV infection is still frequent in high-income countries [16] and especially in low and middle-income countries [17–19]. Although ART is offered free-of-charge in Brazil since 1996, there is a high percentage of late presentation of HIV-infected patients at health services and advanced disease status at the beginning of therapy, as shown in studies conducted mainly before 2010 [20–24]. Guidelines for treating HIV-infected individuals are the same nationally; however, there are differences in health coverage and in the morbidity profile among Brazilian regions [21, 25–27]. Addressing factors associated with the late onset of antiretroviral therapy is a crucial issue in order to end the HIV epidemic, worldwide. It is important to measure and analyze these factors in different epidemiological scenarios. Health care access opportunities and other social, cultural aspects may be associated with the beginning of therapy ART. The aim of this study was to estimate the prevalence of and to investigate the associated factors for late initiation of ART at the baseline of a cohort of ART-naïve patients in a large-sized city in Midwestern Brazil.

## 2. Method

This cross-sectional study was conducted at two public health care units that specialize in treating PLHIV in Goiânia, State of Goias, Midwestern Brazil. Eligibility criteria were age 18 years or older; laboratory diagnosis of HIV infection; no previous use of antiretroviral therapy; enrollment for follow-up and treatment at one of the participating sites; indication to initiate ART for treatment of HIV/AIDS from January 1, 2009 to December 31, 2012. Individuals were treated according to Brazilian guidelines of clinical management of HIV 2008 and 2010 supplement [28, 29]. Briefly, ART was offered to all symptomatic patients regardless of CD4+ cell count values; to all asymptomatic with CD4+ < 200 cells/mm^3^; and for individuals with CD4+ > 200 and ≤ 350 cells/mm^3^ when viral load was higher than 100,000 copies/mL [28]. In 2010, the guideline extended the treatment to all individuals with CD4 < 350 cells/mm^3^ and for those with CD4+ > 350 and ≤ 500 cells/mm^3^ presenting some risk factors such as age older than 55 years, high viral load, cancer, etc. [29].

Data were collected from the Logistics Control of Medications System (SICLOM) of the Brazilian Ministry of Health and from medical and pharmaceutical records. This study excluded pregnant women, patients who did not undergo at least one CD4+ cell count test from 6 months prior to ART initiation to 30 days after initiation, patients with undetectable or absent viral load before the initiation of ART, and those with missing records.

Late initiation of ART was defined as CD4+ cell count < 200 cells/mm^3^ or presence of AIDS-defining illness at treatment initiation or up to 30 days after initiation. This study assessed CD4+ cell counts obtained before the date of first prescription of antiretroviral and the one that was closest to this date. In the case of participants with missing data on CD4+ cell count prior to first prescription of antiretroviral, data from tests performed up to 30 days after first prescription were included in the analysis.

A standardized form was created to collect sociodemographic data (age, sex, skin color, marital status, education level, and place of residence); variables related to the use of health care facility (place of follow-up, number of visits after ART initiation, time elapsed from first HIV-positive result reported in medical records to first visit, and time elapsed from first visit to initiation of ART); clinical and laboratory data (CD4+cell count, pre-ART VL prior, and presence of defining-AIDS illness). Clinical data from this study refer to the period before the initiation of ART. Data were gathered in a database created using Epi-Info®, version 3.5.4, and were analyzed using the Statistical Package for the Social Sciences®, version 21.0 (IBM SPSS Inc., Chicago IL, USA).

Initially, a descriptive exploratory data analysis was performed. Demographic and clinical characteristics of participants were reported as median and interquartile range (IQR) (25^th^ percentile-75^th^ percentile) for continuous variables and as frequency and proportion for categorical variables. Box plots were used to represent the distribution of CD4+ cell count according to the year of initiation of ART and to participants' sexual orientation. Additionally, the prevalence of late initiation of ART and its respective 95% confidence interval (95%CI) were estimated. Categorical variables were compared across PLHIV with late and early initiation of ART using the chi-square test or the Fisher's exact test, as appropriate. The variables of interest included sex, age, education level, place of residence, ethnicity, marital status, sexual behavior, presence of concomitant and opportunistic infections, addictive habits, baseline viral load, pharmacological group of the first antiretroviral regimen, health care facility, and mortality rate.

The magnitude of association between independent variables and late initiation of ART was estimated by adjusted and unadjusted odds ratio (OR) with their respective 95%CI. Variables associated with late initiation of ART, with a significance level lower than 0.20 in univariate analysis remained in the multivariate analysis. The significance level was set at p<0.05.

The Research Ethics Committees of the participating institutions approved the project, under registration number CAAE 36875314.1.3001.0034 and Opinion Number: 1,855,993.

## 3. Results

Overall, 1,371 HIV-infected treatment-naïve participants initiated ART from 2009 to 2012 in the public health system in a large city in central-Brazil. Eighty-two pregnant women were excluded, as well as 148 patients, whose laboratory tests (CD4+ cell count or HIV viral load quantification) did not meet inclusion criteria, resulting in a sample of 1,141 participants. Their age ranged from 18 to 81 years, with a median of 41 years. Patients were predominantly male (69.1%). Regarding sexual behavior, 34.8% of men reported being homosexual or bisexual, 38.2% reported to be heterosexual, and 27.0% of records did not inform sexual orientation. Use of inhaled/smoking drugs (cocaine and crack) and use of injecting drugs were reported, respectively, by 2.5% and 1.4% of the participants.

A total of 637 patients started therapy late, resulting in a prevalence of 55.8% (95%CI: 52.9-58.7). Of these, 555 had CD4+ cell count < 200 cells/mm^3^ and 82 presented with an AIDS-defining illness, although they had CD4+ cell count > 200 cells/mm^3^. Overall, 38.8% of patients had HIV viral load equal to or greater than 100,000 copies/mL.

The median time elapsed from arrival at the specialized health service and initiation of ART was 12 weeks (IQR: 5-88 weeks). As for therapeutic regimen, 68.0% of patients started ART with nonnucleoside reverse transcriptase inhibitors; of these, efavirenz was the most frequently prescribed (98.5%). Protease inhibitors were prescribed for 32.0% of first-line regimens; of these, the most frequent were lopinavir plus ritonavir (LPV/r), accounting for 47.2% of prescriptions, followed by atazanavir plus ritonavir (ATV/r), with 43.1%, and fosamprenavir plus ritonavir, with 10.2%.

Table 1 presents the main sociodemographic and clinical characteristics of the 1,141 participants stratified by late or nonlate initiation of treatment. Participants who started ART late had higher viral load (p=0.004) and used therapeutic regimens containing protease inhibitors more frequently than those who started treatment earlier (p<0.001). There were 65 deaths, resulting in a prevalence of 5.7% (95%CI: 4.5-7.2). Among the patients who died, the median time elapsed from treatment initiation and death was 38 weeks (IQR: 10-103 weeks). There were 35 deaths (53.8%) before 1 year after the initiation of ART. Late initiation of ART was associated with greater risk of death (p<0.001).

Table 2 shows the history of use of alcohol, cocaine, crack, and injectable drugs stratified by late or nonlate initiation of ART. Participants with a history of injecting drug use were associated with late initiation of ART (p=0.01).

In the univariate analysis, late initiation of ART was associated with the following variables: education level, place of residence, color, sex and sexual orientation, hepatitis B virus (HBV) infection, and history of intravenous drug use. Patients who initiated ART late had higher viral load. Additionally, there was also a difference in the choice of antiretroviral regimen between late and nonlate initiation groups. Patients with more advanced immunosuppression received a protease inhibitor drug more frequently compared with those with higher CD4+ cell count. In the adjusted model, late initiation of ART remained associated with lower education level, place of residence, pre-ART viral load higher than 100,000 copies/mL, and HBV infection. Men who have sex with men (MSM) initiated ART earlier compared with women and heterosexual men or with patients lacking information on sexual behavior (Table 3).

**Figure 1:**
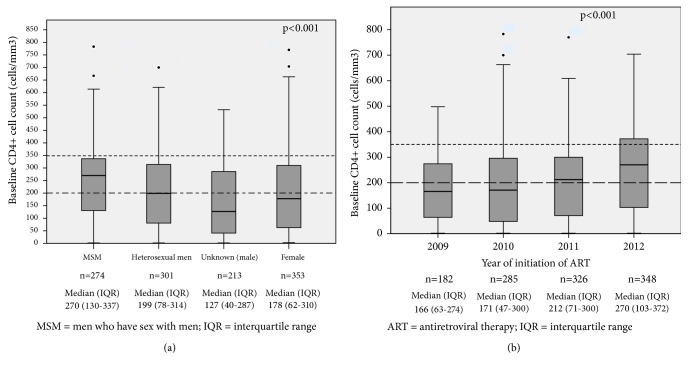
Distribution of absolute values of CD4+ cell count at initiation of ART among people living with HIV or AIDS, according to sex and sexual orientation (a) and year of treatment initiation (b). Metropolitan area of Goiânia, Midwestern Brazil, 2009-2012.

Figure 1(a) represents a box plot for CD4+ cell count considering sexual orientation. The median pre-ART CD4+ cell count was greater among homosexual men (270 cells/mm^3^, IQR: 130-337 cells/mm^3^) compared with values observed in other groups (p<0.01).  Figure 1(b) shows the comparison of CD4+ cell count accounting to year of initiation of ART. Over the 4 years assessed, a progressive annual increase was observed in CD4+ cell count at treatment initiation, with a median of 182 cells/mm^3^ (IQR 63-274 cells/mm^3^) in 2009 and 270 cells/mm^3^ (IQR: 103-372 cells/mm^3^) in 2012 (p<0.01).

## 4. Discussion

We observed a high proportion of late initiation of ART among PLHIV, although these medications have been offered, free-of-charge in Brazil, for almost two decades. Half of our sample initiated ART at an advanced stage of HIV and had a high viral load, which poses a risk of increased morbidity, mortality, and viral transmission. Late onset of ART was associated with lower education level, place of residence outside the metropolitan area and being female. These variables could indicate higher social vulnerability. MSM started ART with higher CD4+ cell count compared with other groups. It may indicate that heterosexual individuals are not yet fully recognized as being at risk, resulting in late HIV diagnosis.

Data from six Latin American countries from 1999 to 2010 revealed that 76% of PLHIV were late initiators of ART, defined as those with CD4+ cell count < 200 cells/mm^3^ prior to ART [17]. A study conducted in Brazil enrolled a large cohort of HIV-infected adults who initiated ART between 2003 and 2010 in four out of five Brazilian regions. The authors found that approximately 60% of PLHIV were severely immunosuppressed, i.e., had AIDS symptoms or CD4+ cell count < 200 cells/mm^3^ when they started ART [21]. It is worth mentioning that Midwestern Brazil, the region where we conducted our study, was not included in that study. However, using the same CD4 threshold we have also found a high frequency of late onset of ART, despite regional disparities. Differently from the mentioned cohort [21], our study included individuals who started treatment before and after adoption of a guideline that expanded the indication for ARV initiation, in 2010 [29]. Despite the high frequency of late onset of ARV, we found a trend towards early initiation after 2010; probably the most important factor is the uniformity of the public health service structure and the adoption of new national guidelines as of this date.

In the present study, low education level and high viral load were associated with late treatment. In general, less educated patients took longer to seek treatment, either because of unawareness of risks or because of difficulties in accessing health services. Low education level is associated with late initiation of ART even where the number of less educated patients is small, such as in high-income countries. A European study assessing a large cohort of patients diagnosed with HIV from 1996 to 2011 found that 40% of participants presented with advanced HIV disease at the beginning of treatment. In that study, the proportion of advanced disease at ART initiation was higher among less educated participants [16].

In our study, MSM initiated ART earlier than women, heterosexual men, and men with missing information on sexual behavior. This result may reflect the greater perceived risk of AIDS for MSM and the consequent seek for early diagnosis and treatment. Nonetheless, our study found that a high percentage of MSM sought health care facilities late, indicating that awareness measures should be further reinforced.

Since viral hepatitis is a condition for early initiation of ART, we did not expect to find that patients with HBV infection had a higher risk for late onset of ART, even after adjusting for some potential confounders such as age and sex. It may be assumed considering our inclusion criteria. Participants should have at least one CD4+ cell count test from 6 months prior to initiation of ART to 1 month after initiation. Therefore, patients who presented with very severe disease may have died before undergoing this test. Nevertheless, the risk of death for late initiators of ART, although possibly underestimated, was significant in the present study. The association of increased mortality rates with delayed diagnosis and late treatment initiation in PVHIV was reported in studies conducted in Brazil [30, 31], low-income countries [32], and high-income countries [33].

There have been improvements in the process of HIV patient care, including a reduction in the proportion of late starters of ART, in many countries [34–36]. Despite these advances, there remain heterogeneity among regions and health care inequities in socially vulnerable groups [37, 38]. Identification of population groups arriving at an advanced stage of the disease at the health service helps in the formulation of public health strategies to promote the early start of ART.

## 5. Conclusions

Despite efforts, the percentage of late initiation of ART is still high, which has important implications for PLHIV and for society. These results showed the need for effective strategies to expand the availability of early diagnosis and treatment of HIV infection, focusing particularly on groups with lower educational level, seeking to overcome the degree of stigma in PLHIV, which restricts their search for health services.

## Figures and Tables

**Table 1:** Baseline characteristics of 1,141 participants, comparing late *versus *nonlate antiretroviral therapy initiation, 2009-2012.

Variables	Total	Late^a^	Non-late	p-value^b^
	n	n (%)	n (%)	
Total	1, 141	637 (55.8)	504 (44.2)	-
Sex				
Male	788	433 (54.9)	355 (45.1)	0.372
Female	353	204 (57.8)	149 (42.2)	
Age (years)				
Median (IQR Q1-Q3)^c^	41 (34-49)	41 (34-49)	40 (33-48)	0.250^d^
Education level				
≤ 8 years	439	269 (61.3)	170 (38.7)	
> 8 years	461	208 (45.1)	253 (54.9)	< 0.001
Unknown	241	160 (66.4)	81 (33.6)	
Place of residence				
Goiânia	697	349 (50.1)	348 (49.9)	
Others	429	281 (65.5)	148 (34.5)	< 0.001
Unknown	15	7 (46.7)	8 (53.3)	
Skin color				
White	180	82 (45.6)	98 (54.4)	
Brown	821	486 (59.2)	335 (40.8)	0.003
Black	51	26 (51.0)	25 (49.0)	
Unknown	89	43 (48.3)	46 (51.7)	
Marital status				
Married/consensual union	286	156 (54.5)	130 (45.5)	
Single/separated/widowed	826	464 (56.2)	362 (43.8)	0.300
Unknown	29	17 (58.6)	12 (41.4)	
Men who have sex with men				
Yes	274	113 (41.2)	161 (58.8)	
No	301	171 (56.8)	130 (43.2)	< 0.001
Unknown	213	149 (70.0)	64 (30.0)	
Viral load (copies/cm^3^)				
< 100,000	698	297 (42.6)	401 (57.4)	<0.001
≥ 100,000	443	340 (76.7)	103 (23.3)	
Hepatitis B virus				
Yes	38	27 (71.1)	11 (28.9)	0.055
No/unknown	1,103	610 (55.3)	493 (44.2)	
Hepatitis C virus				
Yes	20	12 (60.0)	8 (40.0)	0.705
No/unknown	1,121	625 (55.8)	496 (44.2)	
Pharmacological drugs				
NRTI^e^ + NNRTI^f^	777	411 (52.9)	366 (47.1)	0.004
NRTI + PI^g^	364	226 (62.1)	138 (37.9)	

^a^Late initiation defined as baseline CD4+ cell count < 200 cells/mm^3^ or baseline AIDS-defining illness. ^b^Pearson's chi-squared test (p<0.05). ^c^IQR (Q1-Q3): interquartile range (quartile 1- quartile 3). ^d^Student T-test (p<0.05). ^e^NRTI: nucleoside reverse transcriptase inhibitor. ^f^NNRTI: nonnucleoside reverse transcriptase inhibitor. ^g^PI: protease inhibitor.

**Table 2:** Frequency of habits and addiction associated with late initiation of antiretroviral therapy among 1,141 people living with HIV, 2009-2012.

Habits and addiction	Total	Late^a^	Non-late	p-value^b^
	n	n (%)	n (%)	
Alcoholism				
Yes	57	36 (63.2)	21 (36.80)	0.253
No/unknown	1,084	601 (55.4)	483 (44.6)	
Crack/cocaine				
Yes	29	19 (65.5)	10 (34.5)	0.287
No/unknown	1,112	618 (55.6)	494 (44.4)	
Injecting drug use				
Yes	16	14 (87.5)	2 (12.5)	0.010
No/unknown	1,125	623 (55.4)	502 (44.6)	
Other drugs^c^				
Yes	12	9 (75.0)	3 (25.0)	0.179
No/unknown	1,129	628 (55.6)	501 (44.4)	

^a^Late initiation defined as baseline CD4+ cell count < 200 cells/mm^3^ or baseline AIDS- defining illness. ^b^p-value refers to differences between percentage groups with late and nonlate ART initiation by Pearson's chi-squared test. ^c^Marijuana, merla, and heroin.

**Table 3:** Univariate and multivariate analysis of factors associated with late initiation^a^ of antiretroviral therapy among 1,141 people living with HIV, 2009-2012.

Variables	OR (95%CI)	p-value	Adjusted	p-value
			OR^b^ (95%CI)	
Age (years)	1.0 (1.0-1.0)	0.119	1.0 (1.0-1.0)	0.850
Skin color				
Brown	1.7 (1.2-2.4)	<0.001	1.4 (1.0-2.0)	0.082
Black	1.2 (0.7-2.3)	0.299	1.2 (0.6-2.5)	0.621
Unknown	1.1 (0.7-1.8)	0.383	0.7 (0.4-1.2)	0.173
White	1		1	
Education level				
Unknown	2.4 (1.7-3.3)	<0.001	2.0 (1.4-2.5)	<0.001
≤ 8 years	1.9 (1.5-2.5)	<0.001	1.7 (1.2-2.5)	0.001
> 8 years	1		1	
Place of residence^c^				
Others	1.9 (1.5-2.4)	< 0.001	1.4 (1.1-2.0)	0.003
Goiânia	1		1	
Marital status^d^				
Single/separated/widowed	1.1 (0.8-1.4)	0.341	-	
Married/consensual union	1			
Sex/Sexual behavior				
Female	2.0 (1.4-2.7)	< 0.001	1.7 (1.1-2.5)	0.016
Heterosexual male	1.9 (1.3-2.6)	< 0.001	1.4 (1.0-2.5)	0.030
Male/Unknown sexual behavior	3.3 (2.3-4.8)	< 0.001	2.5 (1.7-5.0)	< 0.001
Homosexual/Bisexual male	1		1	
Injecting drug use				
Yes	5.6 (1.4-36.8)	0.010	3.3 (0.7-10.0)	0.133
No/unknown	1		1	
Viral load (copies/cm^3^)				
≥ 100,000	4.4 (3.4-5.8)	< 0.001	5.0 (3.3- 10.0)	< 0.001
< 100,000	1		1	
Hepatitis B virus				
Yes	2.0 (1.0-4.2)	0.039	2.5 (1.25-5.0)	0.013
No/unknown	1		1	
Hepatitis C virus				
Yes	1.2 (0.5-3.1)	0.439	-	
No/unknown	1			
Pharmacological drugs^e^				
NRTI + PI	1.5 (1.1-1.9)	0.002	1.4 (1.0-1.7)	0.041
NRTI + NNRTI	1		1	

^a^Late initiation defined as baseline CD4+ cell count < 200 cells/cm^3^ or baseline AIDS-defining illness. ^b^OR = odds ratio; 95%CI = 95% confidence interval. ^c^Missing information: n=15. ^d^Status marital: n=29. ^e^NRTI: nucleoside reverse transcriptase inhibitors. Regression model adjusted for age, skin color, education level, place of residence, sex/sexual behavior, injecting drug use, viral load, hepatitis B virus, and pharmacological drugs group. NNRTI: nonnucleoside reverse transcriptase inhibitor; PI: protease inhibitor.

## Data Availability

The data used to support the findings of this study are restricted by the Brazilian Ethics Committee in Research (n.510/2016) in order to protect patient privacy. Data are available from Priscila Pacheco (prprirgp@gmail.com) for researchers who meet the criteria for access to confidential data.
